# Metal-Organic Frameworks as Multifunctional Materials for Triboelectric Nanogenerators Applied in Self-Charging Power Sources

**DOI:** 10.1021/acsomega.6c03646

**Published:** 2026-06-16

**Authors:** Helinando Pequeno de Oliveira

**Affiliations:** Instituto de Pesquisa em Ciência dos Materiais, Universidade Federal do Vale do São Francisco, 48902-300 Juazeiro, BA, Brazil

## Abstract

The singular porous structures of metal–organic frameworks (MOFs), with metal ions or clusters as central nodes and organic compounds as ligands, offer a wide range of environmental and energy-based applications. In particular, for prototypes of triboelectric nanogenerators, the versatile role of MOFs enables their use as friction-layer nanofillers and a means to tune the dielectric constant of tribopairs, thereby improving surface charge activity for both tribopositive and tribonegative layers. The functionalization of MOFs with standard electron-donating and -withdrawing groups and the production of composites improve both tribopairs abilities and the device’s performance under high-humidity and high-temperature conditions. Also, strategies for faster charge-transfer rates in self-charging power sources are discussed, with a view to integrating MOF-based supercapacitors into all MOF devices that leverage the outstanding properties of these structures. In this Review, a comprehensive evaluation of strategies for using MOFs and their composites as nanofillers in tribopairs is provided, with a focus on the potential-well interpretation of doping processes in tribopairs and their impact on the overall performance of energy-harvesting devices and the extension in the limit of operation at high relative humidity, characterizing a critical drawback in TENGs based on standard tribolayers and for integrated devices in which the power output at interfaces can be controlled.

## Graphical abstract

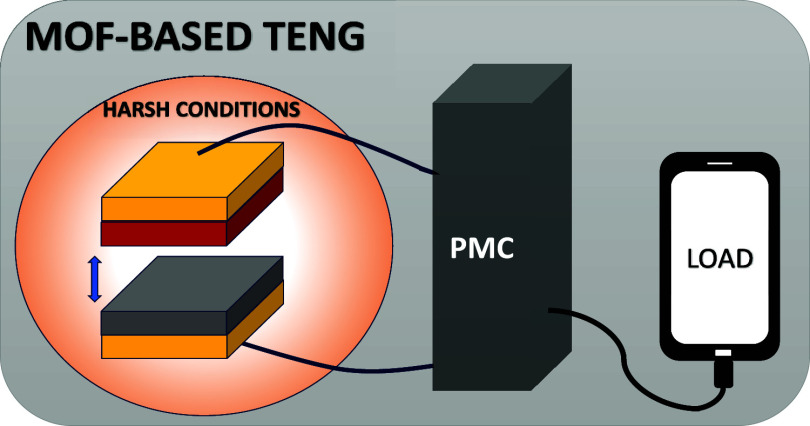

## 1 Introduction

The low cycle life, continuous recharging, and limited integration of conventional batteries with wearable electronic devices and smart textiles underscore the need to develop sustainable, autonomous (self-powered)^1,2^ energy sources.^3^ Energy harvesting systems are promising candidates for developing future battery-free devices, in which biomechanical energy (from wind/rain or body movement (walking, running, breathing, or touching))^4,5^ can be harvested and converted into electrical energy.^6^ Despite their common occurrence in daily life, the effects of triboelectric charging were not realized in devices until 2012, when a group led by Zhong Lin Wang first reported experiments on triboelectric nanogenerators (TENGs).^7^ These devices can convert high-entropy energy into an AC electric signal by using sustainable principles. As a consequence, TENGs have attracted considerable interest in the literature due to their promising applications in energy source development, the Internet of Things, and environmental sensors,^8^ based on a diversity of available operating modes and low-cost materials that can serve as tribopairs,^6,9^ being subjected to triboelectrification (contact electrification) and electrostatic induction.^3^

These processes are favored by periodic contact-separation steps of the tribopairs, in which electrostatic charges are induced by contact between the surfaces of the tribopositive and tribonegative layers. Under the tribolayer separation, an electric potential is generated, enabling an electric current to flow toward the external circuit. The theoretical approach underlying the overall process of charge generation in TENGs differs from the conventional overview, which considers fixed dielectric-layer (tribopairs) boundaries and volumes. The newly defined displacement vector (see eq 1) receives an additional term (**P**_S_), since the electrostatic charges created by the triboelectric effect cannot be represented as free charges (which are represented by the term ε_0_E), nor by local polarization **P** (created by electric field **E**), being created by the mechanical movement imposed on the surfaces.

$$D=\varepsilon_{0}E+P+P_{s}=D^{\prime }+P_{s}$$ 1

Based on this extra term, Wang proposed the expanded form of Maxwell’s equation (see eqs 2–5, in which ***B*** represents the magnetic field, ***E*** is the electric field, ***H*** is the magnetizing field, and ***J***_***F***_ represents the free-flowing current density—the conduction current provided by the nanogenerator).

$$\nabla \cdot D^{\prime }=\rho_{F}-\nabla \cdot P_{s}$$ 2

∇·B=0 3

$$\nabla \times E=-\left(\frac{\partial }{\partial t}+v\cdot \nabla \right)B$$ 4

$$\nabla \times H=J_{f}+\left(\frac{\partial }{\partial t}+v\cdot \nabla \right)(P_{S}+D^{\prime })$$ 5

The addition of an extra term (the polarization term (***P***_***s***_)) in the displacement vector characterizes a time-dependent term in which the movement of dynamically charged media is conveniently represented, physically manifested by the appearance of charges due to the triboelectrification,^7^ with effects on the total displacement current that characterizes the current observed in a nanogenerator, represented by eq 6

$$J_{D}=\frac{\partial D^{\prime }}{\partial t}+\frac{\partial P_{s}}{\partial t}=\varepsilon \frac{\partial E}{\partial t}+\frac{\partial P_{s}}{\partial t}$$ 6

The second term in eq 6, referred to as the Wang term,^10^ characterizes the device’s current contribution due to external mechanical excitation. As a result, the total current in the system is given by eq 7

$$J_{T}=J_{f}+\rho_{f}v+J_{D}$$ 7

As a consequence, the expanded Maxwell′s equations incorporate the tribolayer velocity term into the overall response. In general, the overall performance of TENG (measured by power density, short-circuit current, and open-circuit voltage) is primarily determined by the surface charge density^6^ and the work function differences between the tribolayers. The relationship between the maximum charge density in a contact-separation TENG (with a parallel plate configuration) is given by eq 8 ^11^

$$\sigma^{\prime }=\frac{\sigma_{0}x\left(t\right)}{x\left(t\right)+d/\varepsilon_{r}}$$ 8

where σ′ is the maximum charge density, σ_0_ is the charge density at the equilibrium, *x*(*t*) is the distance of tribopairs for the contact-separation configuration, *d* is the thickness, and ε_r_ is the dielectric constant of the tribonegative layer. In addition, both short-circuit current and open-circuit voltage vary directly with σ′.^11^ From this relationship, it is possible to observe that an increase in the dielectric constant of tribopairs enhances the output performance of the resulting TENG.

The standard ranking of materials in the triboelectric series, based on their ability to repel or attract electrons, is a starting point for selecting materials for assembling TENGs, with metals (copper, aluminum) considered as standard electron-donating systems (tribopositive materials). At the same time, fluorinated polymers are tribonegative species (electron trapping systems). Despite the combination of extreme tribopairs in the triboelectric series, the device’s bottleneck remains its low power output, due to low short-circuit current, high internal impedance, and structural instability. To circumvent this drawback, three important strategies have been considered in the material engineering:

i) The material surface modification with friction layer additives;
ii) The incorporation of nanofillers into the tribolayer;
iii) The improvement in the dielectric constant of the tribolayer.

Therefore, the most common strategies to optimize device performance focus on controlling the dielectric constant of tribolayers through functionalization and filler incorporation, with the effects on surface roughness.^12^ The incorporation of nanofillers into dielectric layers has been explored to control the surface area, crystallinity, and porosity of the resulting material,^13^ with direct consequences for induced and trapped triboelectric charges^14^ by reinforcing interfacial polarization,^15^ which is affected by the chemical functionalization of the species. On the other hand, the distribution of nanofiller sizes affects the roughness of the resulting dielectric layer.^16^ In summary, these modifications must be concentrated in four structures: the charge-generating layer, the charge-trapping layer, the charge-collecting layer, and the charge-storage layer. The primary source of improved charge-generating effectiveness is the controlled area enabled by the assembly of micro/nanoscale structures, with direct implications for the number of generated charges, with secondary control via dielectric constant. Regarding the charge-trapping layer, it avoids that charge-generation step can be achieved by combining the surface with oppositely charged induced species, thereby reducing the TENG performance (i.e., the triboelectric potential).^17^ The most common fillers for dielectric layers in TENGs are lignin,^18^ cobalt aluminum layered double hydroxide,^19^ 2D carbon nanofillers such as graphite, graphene oxide, and graphene nanoribbons,^20^ inorganic fillers (TiO_2_, SiO_2_, BaTiO_3_, ZnSnO_3_, MoS_2_),^21^ 2D nanofiller MXene (Ti_3_C_2_T_*x*_), hexagonal boron nitride nanosheets (hBNNs), and reduced graphene oxide (rGO),^22^ polymorphic formamidinium lead triiodide (FAPbI_3_) perovskite nanofillers,^23^ and metal–organic frameworks (MOFs).^24^

Metal organic frameworks (MOFs) are metal ions or clusters bonded by organic linkers, resulting in 1-D to 3-D structures that have attracted attention in the literature due to their impressive properties (large surface area in the range of 1000–10,000 m^2^/g, porosity greater that 50% of the MOF crystal volume and tunable pore size (in the range of 0–3 nm),^25^ and flexible tailorability in tunable structures). The most common types of MOFs are Isoreticular MOFs, zeolitic imidazolate frameworks (ZIFs), porous coordination networks (PCNs), Materials Institute Lavoisier (MIL) MOFs, porous coordination polymers (PCPs), and University of Oslo (UiO) MOFs, with more than 120,000 experimental crystal structures of MOFs in the Cambridge Structural Database (CSD).^26^ In addition to pristine MOFs, multifunctional components leverage interactions among MOFs and metal oxide nanoparticles, graphene oxide, MXene, polymers, and silica.^27^ The interaction reinforces the electrical conductivity, pH stability, and recyclability of MOFs, as part of general strategies ranging from defect engineering and heteroatom doping to providing outstanding properties for MOF-based composites. As a consequence, potential applications of MOF-based materials are explored across a wide range, including catalysis, drug delivery, gas storage and separation, biomedical and environmental applications, energy storage, and quantum-related applications.^25,27–29^

The increasing interest in MOF-based systems in the literature is evident from Figure 1a, which shows a sharp increase in the number of papers considering MOFs over the last 20 years. Regarding the use of MOFs for TENG-related applications, an increase in the number of papers is also observed in the 2020–2026 period (Figure 1b). It is worth mentioning that another exponential growth is observed for self-powered sensors in recent publications (Figure 1c) and the relative percentage of TENG-based devices applied in self-powered sensors (see Figure 1d), with 22% of applications in sensors provided by TENGs. These data reinforce the trend for the use of MOFs in self-powered sensors and related devices via TENGs.

**Figure 1:**
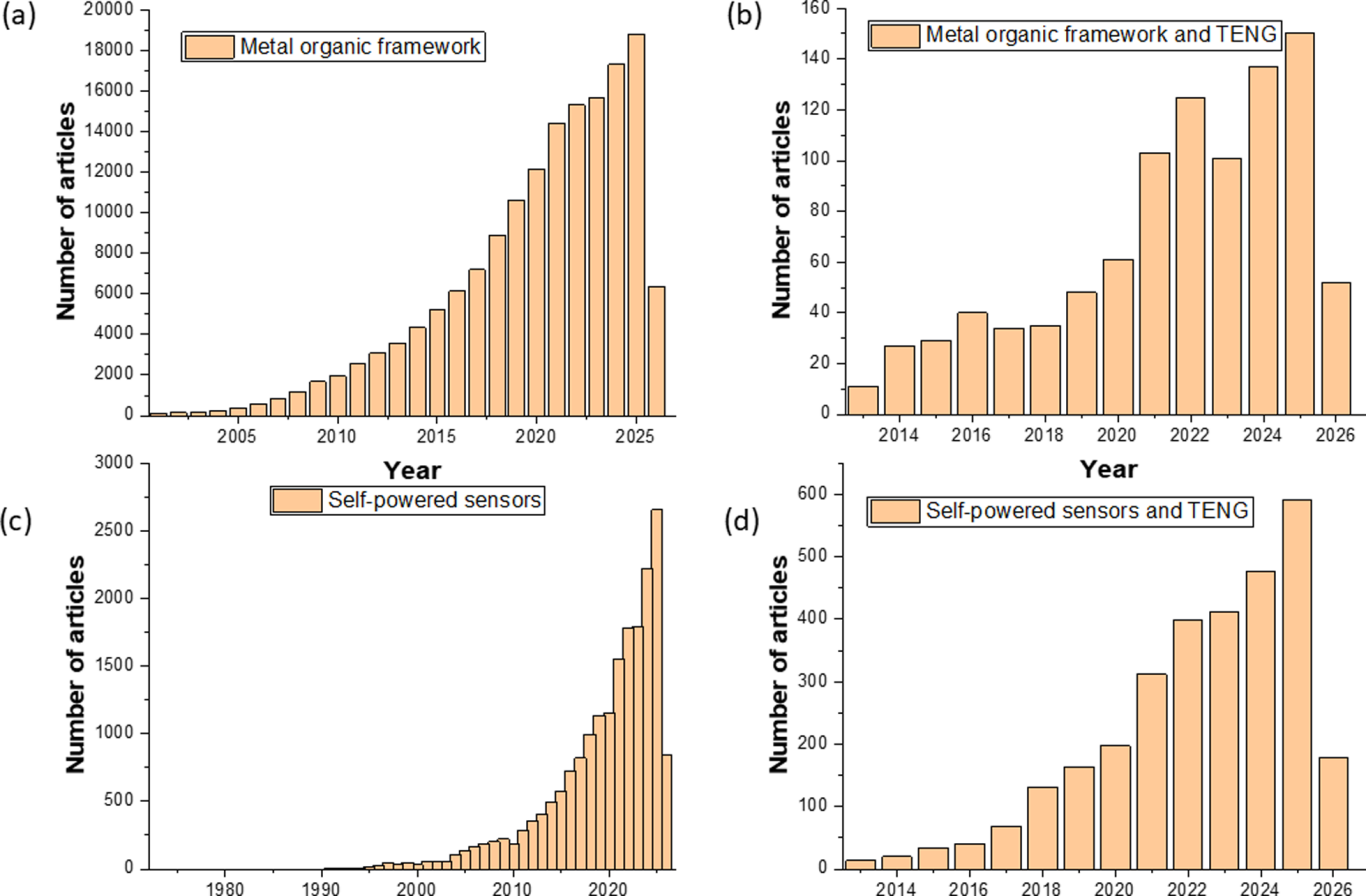
Number of published papers in the Web of Science platform (verified at 05/09/2026) for (a) term: metal organic framework, (b) term: metal organic framework and TENG, (c) term: self-powered sensors, (d) term: self-powered sensors and TENG.

For MOF-based TENGs, in addition to the applications involving the detection of acceleration, pressure, strain, and temperature in human intelligent monitoring systems,^30^ sensors for the detection of force and humidity were provided by the use of ZIF-8-CF_3_/PVDF-based TENG.^24,31^ Ma et al.^32^ reported the use of a spiky structured ZIF-8@ZnO operating as a conventional TENG and also as a self-powered methanol sensor. The porous structure of ZIF-8@ZnO enables the detection of methanol at 100 ppm. Han et al. proposed a nitrogen-doped metal–organic framework-derived In_2_O_3_ sensor with a limit of detection of 1 ppm NO_2_ at room temperature.^33^ In general, it is considered that the participation of MOFs in two critical processes involves inducing charges and trapping them. In general, charge traps are observed in physical defects in insulators, whereas for MOFs, fluorine-modified groups are reported to increase trapping ability.^34^ Chen et al. used scanning tunneling microscopy and spectroscopy to visualize substrate charges trapped beneath the pores of two-dimensional conjugated metal–organic frameworks.^35^

As observed, the rich pore chemistry of MOFs (pore structure and pore size) significantly influences the overall electrical performance of the derived TENGs, as their structures can accommodate a high density of charges. Upon compression, the MOF-based porous structure exploits the electrostatic induction effect to generate additional charge, which is accommodated on its porous surface, enhancing the charge density.^36^ Xi et al. reported the influence of ZIF-67 grain size on the resulting dielectric constant of the tribolayer, observing that a decrease in MOF particle size leads to greater charge-trapping capacity,^37^ which has been considered in the literature as an active strategy to reach a superior surface charge density.^38^ Venkatesan et al. described the direct relationship between the structure of MOF and superior electrostatic induction effects by the optimization in the gap between the primary electrode and the charge trapping-layer provided by a defective and porous dielectric layer.^39^

For energy-storage-related applications, three of the most important properties to be considered in TENGs are the large surface area, remarkable porosity, and tunable structure, enabling several functionalities.^40^ Important drawbacks for energy-related applications are the electrical conductivity (relative to highly conductive materials such as graphene and graphite) and the need to meet stability. With this aim, high-valence metal sites reinforce the overlap of the metal–ligand orbitals, as observed in Fe-based MOFs.^41,42^

In particular, MOFs play a pivotal role as emerging materials for several applications due to their singular structure, such as uniform cage-like pores from zeolitic imidazole frameworks—ZIFs (a subclass and the most studied subclass of MOFs for TENG applications)^43,44^ with multiple crystallinities,^45^ in highly ordered structures composed of ligand–metal ions/clusters with organic ligands through coordination bonds^34^—in which is observed characteristic high density of charges disposed on rough surface.^46^ Resulting structures are characterized by high surface area, high porosity, and chemical reactivity, enabling applications in several areas such as charge storage, catalysis, gas storage, and self-powered applications—energy harvesting in TENGs. Pristine MOFs, as triboactive materials, are favored by their surface properties^47,48^ which enable the direct disposition on electrodes (such as the adhesive side of copper tape)^44^ or deposition as nanocrystals of ZIF-8 on the surface of polyacrylonitrile (PAN) nanofibers.^49^ Despite the advantage of direct application as a friction layer, there are issues regarding limited stability and lifetime under repeated use.^44^

A promising strategy that circumvents these drawbacks is the use of MOFs as nanofillers in standard polymeric friction tribopairs, potentially favored by their unique properties, which enable mutual tuning of the dielectric constant, surface charge density, and surface roughness^5,13–16,50–52^ with the polymer′s electron-gaining and withdrawing properties being reinforced by the hierarchical structure of MOFs.^53–57^

In this review, the influence of MOFs on the resulting performance of TENGs is discussed in view of the strategies for enhancement of dielectric properties and/or surface modification (porosity/ surface area) of the tribolayer, its influence on the hydrophobic behavior, and the humidity role (harsh conditions) on the energy conversion degree. The development of MOF-based composites is also evaluated for friction properties under external factors, such as water and oxidative conditions, to assess the overall performance of the assembled TENG. Drawbacks of lifetime and continuous operation as a friction layer are discussed in light of the requirements for power robustness and energy harvesting efficiency. These strategies are conveniently explored for use in self-charging units by effectively integrating MOF-based supercapacitors to meet the adequate requirements for portable electronics.

## 2 MOFs as Multifunctional Nanostructures for TENGs

The electrical output performance of TENGs depends on their ability to donate or accept electrons.^47^ Standard tribonegative materials (such as fluorinated ethylene propylene (FEP), polyvinylidene fluoride (PVDF) and polytetrafluoroethylene (PTFE)) are fluorene-based compounds, indicating that functionalities are critical to reinforce both electron-donating abilities (−CH_2_, −OH, −NH_2_ groups) and electron withdrawing ability (−NO_2_, −Br groups) being able to reduce the electron density in π systems, enhancing their electronegativity, as a consequence of the reduction in the lowest unoccupied molecular orbital (LUMO) level and reinforcing the capture and trapping of the incoming electron along with the contact of different tribolayers.^58,59^ Wang et al.^53^ reported that strong withdrawing groups in PDMS, used as reinforcement additives, increased the surface charge density and, as a result, the power density by a factor of 77.5. Results summarized in Figure 2 confirm the effective role of MOF (UiO-66-4 F) incorporated as a filler in PDMS, which presents (in a pure state) an average surface potential of −1.4 V (see Figure 2A), while the doped PDMS (UiO-66-4 F@PDMS) presents a significant increase in the voltage average (*V* = −6 V), as shown in Figure 2B which is in agreement with the corresponding topography in Figure 2C,D characterizing higher rugosity degree for doped PDMS. The corresponding variation in the dielectric constant as a function of mass ratio of UiO-66-4 F and PDMS confirms a general increase in the response with a well-defined maximum at 9% of MOF filler (Figure 2E), with the corresponding distribution of surface potential (Figure 2F) confirming the contribution of MOF as a filler for PDMS, reinforcing its tribonegative behavior in overall response.

**Figure 2:**
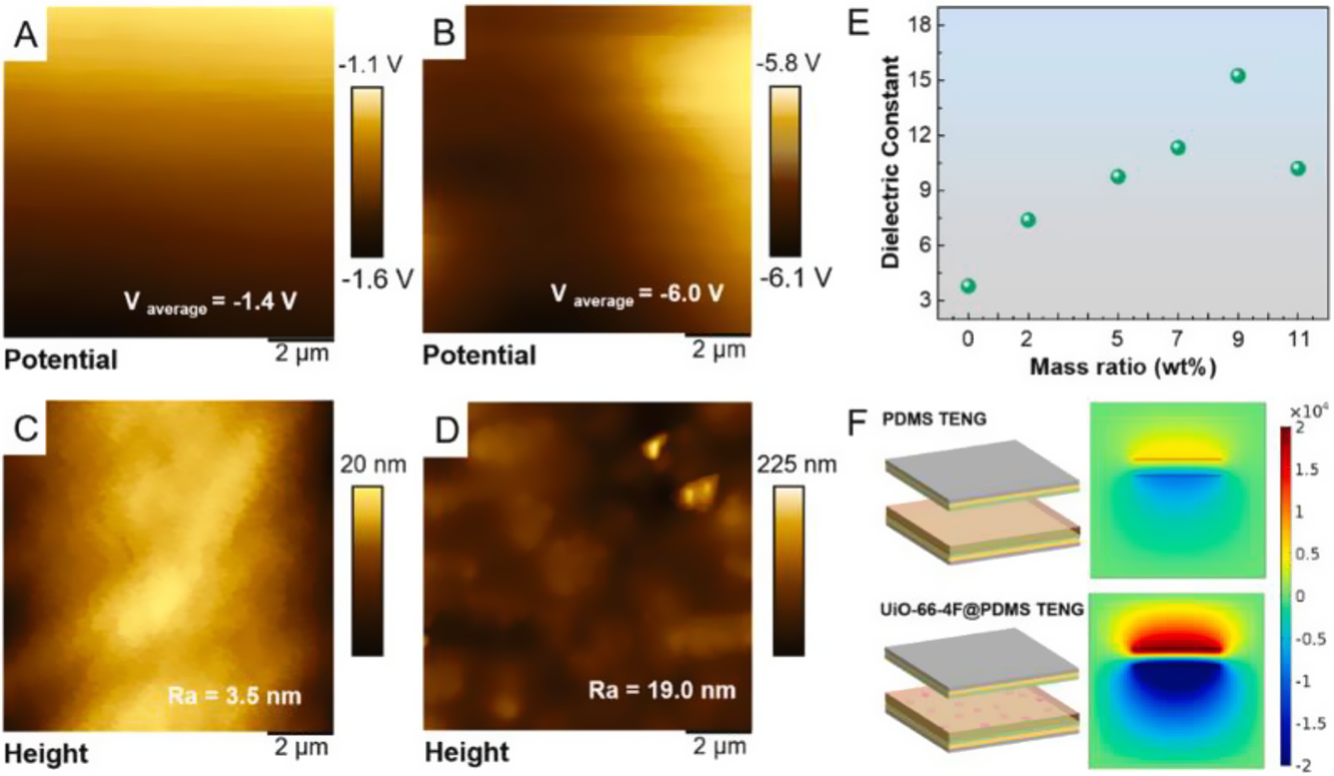
Surface potential profile for PDMS film (A) and UiO-66 4 F@PDMS film (B); AFM topography for PDMS film (C) and UiO-66 4 F@PDMS film (D); corresponding dependence of the dielectric constant at varying mass ratio of UiO-66 4 F (E), and (F) simulated surface potential distribution for assembled device. Reprinted from Nano Energy, 107, Y.-M. Wang, X. Zhang, C. Liu, L. Wu, J. Zhang, T. Lei, Y. Wang, X.-B. Yin, R. Yang, Remarkable improvement of MOF-based triboelectric nanogenerators with strong electron-withdrawing groups, 108149, Copyright (2023), with permission from Elsevier.

It is worth noting that MOFs can act as positive and negative tribolayers under appropriate functionalization, as observed for fluorinated MOFs, which are considered to be standard tribonegative materials.^60^ The reversal of the charge polarity in MOFs is primarily affected by the ligand groups attached to the structure. As a consequence, the resulting optical band gap of the MOF, together with its comparison with the corresponding value for the tribopair, defines the prevailing polarity of the material. For cases in which the bandgap of the MOF is higher than that of the corresponding polymeric layer, the preferred electron transfer is from the polymer LUMO to the MOF conduction band (which acquires the characteristic of a tribonegative material). On the other hand, by combining a lower band gap MOF with a higher band gap polymer layer, the flux of electrons tends to be from the conduction band of the MOF to the HOMO of the polymer layer (the MOF acquires the characteristic of a tribopositive material). An interesting application of MOF tunability in TENGs is the reversal activity of polyacrylonitrile (PAN) polarity upon the incorporation of different MOFs into polymeric solutions used in electrospinning assays. In this context, electrospun PAN/ZIF-8 and PAN/MIL-100 fibers have been reported for all-PAN fiber TENGs based on the same polymer template, exhibiting promising humidity-dependent responses,^61^ thereby enabling their use as humidity sensors. The corresponding evaluation of the tribopolarity of the MOFs was performed using Kelvin probe force microscopy (KPFM), a convenient tool for measuring the sample’s work function (which is proportional to the measured voltage). Positive potential measured in KPFM reflects a material’s tendency to attract electrons (characterizing a promising tribonegative material). The authors provided a classification of MOFs by KPFM-measured voltage, ranking the materials from most tribonegative to most tribopositive.

In addition to the reversion in polarity induced by functionalities in MOFs measured from the electrostatic surface charge structures, there is another critical issue to be considered in applications, which regards the durability/ stability of the tribolayer under continuous abrasive conditions of use.^31^ This aspect is critical for the prevailing application of MOFs as nanofillers in tribonegative layers of TENGs, as discussed in the following section.

### 2.1 MOF as Nanofillers for Tribonegative Layers of TENGs

The advantage of using MOFs and functionalized MOFs in polymeric templates with high electron-gain capability (such as PDMS and Ecoflex) lies in the tunable dielectric constant and improved surface adhesion afforded by their multifunctional mesoporous structures, as described in ref 62 for the impregnation of ZIF-71 and ZIF-72 into PDMS and for incorporation into the Ecoflex layer, exploring the high porosity and characteristic charge trapping of the MOF.

A comprehensive discussion about reinforcement mechanisms in TENG performance, arising from the action of MOF and doped MOF within a polymeric tribonegative layer, is provided by Wen et al.,^58^ who evaluated the role of different functionalities in the overall electrical performance of the resulting TENG using Cu and PDMS as standard tribopairs. With this aim, the authors studied the influence of devices functionalized by an electron-withdrawing group -NO_2_ and -Br, and with an electron-donating group -NH_2_.

Results summarized in Figure 3 for output voltage (a), current (b), and transfer charge (c) and normalized variation (d), confirming the best electric output performance for electron-withdrawing groups in opposition to that observed for electron-donating group −NH_2_, following the order PDMS@UiO-66-NO_2_ > PDMS@UiO-66-Br > PDMS@UiO-66> PDMS@UiO-66-NH_2_ > PDMS, confirming the dominant role of PDMS as tribonegative layer and the adequate modification provided by corresponding functional groups. The interpretation provided by the authors considers a potential-well model in which *E*_cu_ and *E*_P_ represent the occupied energy levels of electrons in the atoms of the tribopairs in the tribopair formed by PDMS and Cu, while *E*_1_ and *E*_2_ denote the potential energy levels for electron escape from the Cu surface into the PDMS. Upon contact between the parts and overlap of the electron clouds, electrons migrate from the copper layer to the PDMS layer. Under modification with MOF as a nanofiller (UiO-66), the energy level E_P_ decreases to *E*_P1_, and under functionalization with (−NO_2_), a lower energy level *E*_P2_ is observed in a new configuration in which more electrons can be transferred from Cu to PDMS@UiO-66-NO_2_ in comparison to the Cu to PDMS@UiO-66 and PDMS, respectively, resulting in a higher electron transference ratio, implying a highly efficient TENG, as confirmed by the measured electrostatic surface potential.

**Figure 3:**
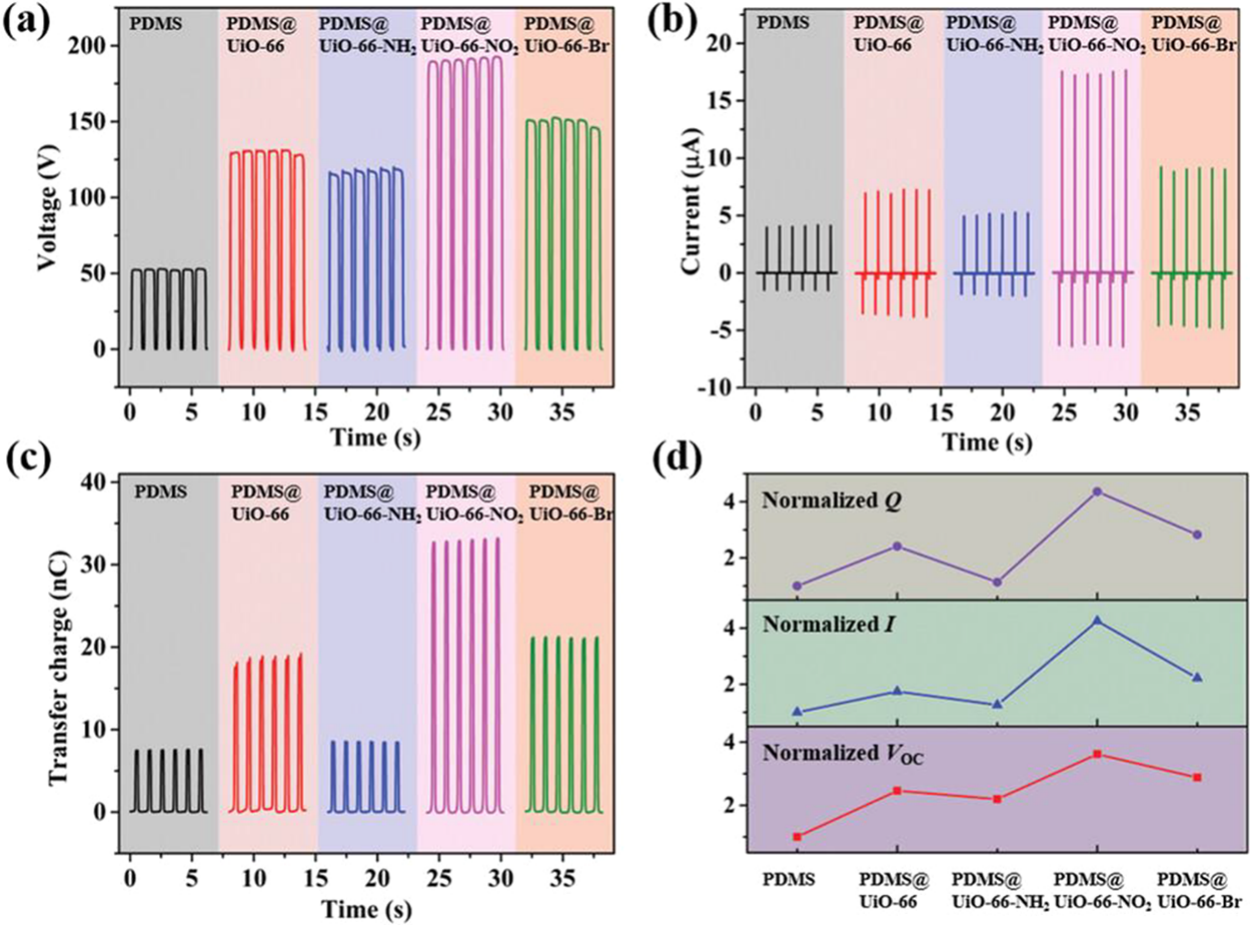
Plots of output voltage (a), current (b), transfer charge (c), and normalized TENG output for doped PDMS tribolayer (d). Reprinted with permission from R. Wen, B. Zhao, L. Fan, J. Guo, J. Zhai. Controlling the Output Performance of Triboelectric Nanogenerator Through Filling Isostructural Metal–Organic Frameworks With Varying Functional Groups. *Adv. Mater.*, Copyright [2023] John Wiley and Sons.

In addition to the increase in the charge density for transference upon contact, there are several aspects involved in the improvement of the TENG output performance, such as uniform porosity and chemical stability of MOFs disposed in a hybridized assembly of MOF525@silicone@α-MoO_3_ nanocomposites assembled as a single electrode TENGs operating with human skin as a tribopositive pair.^63^ Similarly, multilayers of Ecoflex were assembled with MOF-525 and cobalt-nanoporous carbon@MXene, facilitating ion transport in the bulk of the structures and creating charge traps.^64^ Rahman et al.^65^ reported the use of MOF-derived cobalt-based nanoporous carbon incorporated into PVDF as a nanofiller, as part of a strategy to enhance the dielectric constant of the PVDF layer and promote the formation of the electroactive β-phase in PVDF, thereby achieving impressive performance at high relative humidity. The data on TENG performance under harsh conditions (such as high humidity) indicate a dramatic decrease in energy harvesting due to the formation of a water layer on the tribolayers, which critically affects the charge-generation step. Wen et al.^66^ reported a MOF-based TENG produced by incorporating HKUST-1 (Cu_3_(BTC)_2_) into a polydimethylsiloxane matrix. The authors observed an increase in the device’s performance at higher relative humidity and attributed this to the adsorption and desorption of water molecules in the nanochannels of HKUST-1. This process is achieved by increasing the material’s electron-trapping capacity, suggesting that surface triboelectric electrons are captured within the frictional layer. Corresponding functional modifications are also provided for tribopositive layers of TENGs, as follows.

### 2.2 MOF as Tribopositive Layers of TENGs

Despite the dominant efforts that have been focused on the tunability of tribonegative polymers, by the impregnation of nanofillers in typical tribonegative support systems, the investigation about tribopositive layers remains necessary since there are issues to be considered in the improvement of positive tribolayers for TENGs: unmodified metal layers (Cu or Al) raise concerns about comfort and wearability with oxidative issues to be considered under direct contact with the skin in body movement sensors. These drawbacks underscore the need for research into new tribopositive pairs that meet the requirements of efficiency in charge trapping and transfer and wearability. Following the correspondence from the successful strategy for incorporating electron-withdrawing groups into tribonegative materials, Xu et al.^59^ proposed modifying UiO-66 with electron-donating groups (−H, −CH_3_, −NH_2_, and −OH) as a tuning strategy to control the tribopositive degree of the polyurethane. KPFM measurements returned the potential difference between the tip and tribolayer and returned a negative value for PDMS (−2.25 V) with positive ones for PU derivative layers following the order (−OH > −NH_2_ > −CH_3_ > −H) as indicated in maps from Figure 4a, which introduces a schematic representation of the surface state model (Figure 4b) at separation and contact of layers. Under contact, electrons tend to be transferred from the material with a higher surface state to that with a lower surface state. The decreasing work function observed in Figure 4b leads to an increased band gap, which is reflected in a higher electron transfer ratio toward the PDMS. DFT evaluation by the authors (Figure 4c) confirmed that the highest occupied crystalline orbital is located on the -OH group, which is characterized as an active charge-transfer site (see Figure 4c,d).

**Figure 4:**
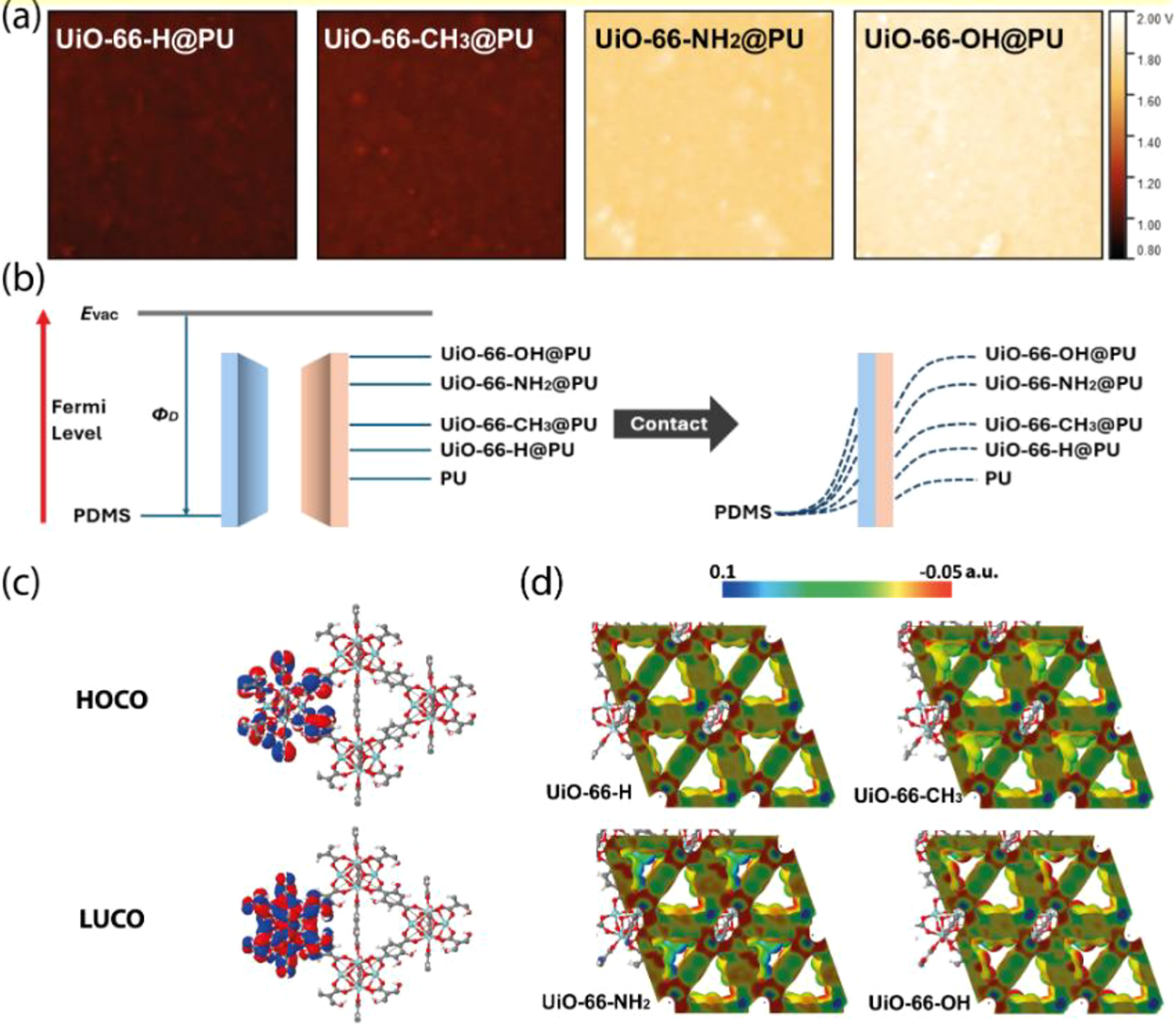
(a) Comparison of surface potential of UiO-66-X@PU films from KPFM measurements, (b) Effect of doping level on surface state model of tribopairs, (c) HOCO and LUCO crystal orbitals for UiO-66-OH, and (d) potential maps calculated by DFT of the four different UiO-66-X frameworks. Reproduced from ref 59. Published under a Creative Commons CC-BY 4.0 license.

Alternatively, tribopositive properties for MOF-based compounds have been explored from the direct impregnation of materials on substrates, such as carbon clothes,^67^ by their growth on indium tin oxide-polyethylene terephthalate (ITO-PET),^68^ or using electrospun fibers loaded with MOF precursors, in which the resulting nanofibers act as microreactors for the growth of microcrystals of MOFs.^69^ The impregnation of MOFs on electrode surfaces (such as Al or Cu tape) is characterized by poor adhesion under abrasive conditions.^70^ Strategies for reinforcing mechanical and environmental stability involve chemical surface modification to achieve desirable superhydrophobic properties, such as modifying cotton fabric with polydopamine and treating it with hexadecyltrimethoxysilane, thereby improving performance under high-humidity conditions.^71^ Jayababu et al. reported the use of a bimetallic organic framework (Co/Zn) coated on flexible conductive fabric (FCF), considered a tribopositive pair, combined with PTFE as a negative tribolayer.^72^ The use of cyclodextrin as a ligand and MOF exploring sodium as a metal ion behaves as a tribopositive layer of TENG that, in combination with the Teflon pair, returned an electrical output of 152 V and 1.2 mA for a TENG of α-CD MOF/Teflon pair.^64^ In particular, ZIFs are promising tribopositive pairs that have been explored in combination with Kapton as negative tribolayers.^73^ In addition to the functionalization of MOF derivatives applied as nanofillers, composites with MOFs are important building blocks in which electrical transport favors the charge transfer, as discussed in the following section.

### 2.3 MOF-Based Composites for TENGs

MXenes are 2D transition-metal carbides or nitrides with characteristic high electrical conductivity, dense surface functional groups, mechanical flexibility, and high surface area,^74–80^ opening several possibilities for use in TENGs as frictional layers, fillers, and electrodes.^80^ In addition, the presence of both electron-accepting fluorine and electron-donating oxygen atoms enables the use of MXene as a tribopositive or tribonegative material.^81^

Despite these outstanding properties, there are critical issues to be addressed for the use of MXene derivatives in TENGs, including oxidation of the material surface upon exposure to water and oxygen, as well as restacking of MXene sheets. In both conditions, the in situ growth of MOFs on the surface of MXene lamellae avoids the contact of MXene with water molecules due to the MOF micropores^82^ and by the intercalation of MOFs between MXene nanosheets, improving the stability of the composite.^83,84^ Regarding the use of MOF as filler for tribonegative layers, it has been reported that MXene incorporated into silicone has been considered as a reinforced electron-accepting layer,^80^ while tribopositive films of MOF808@MXene are paired with PVDF, generating an electrical output performance of 96.22 V, 30.7 μA, and 67.4 mWm^–2^.^47^ It is worth mentioning that TENG’s current is displacement-driven rather than conduction-driven. As a consequence, the high electrical conductivity of nanofillers (MXene, MOFs, graphene, and conducting polymers) boosts the efficiency of TENG due to the acquired ability of the material to attract and trap electrons.^81^

On the other hand, the use of ZIF-8 and MXene as fillers for PDMS reinforced the application in droplet energy harvesting.^82^ In this work, Wang et al. reported the presence of dangling Zn ions on the MXene surface, thereby optimizing surface hydrophobicity, which is critical for the reported application, in which a droplet slides off the friction layer to harvest energy in a TENG^82^ prototype. As summarized in Figure 5a, interdigitated electrodes (in yellow) are critical for optimizing energy conversion in the overall process, in which the first electrode acquires a negative charge due to direct contact with the positively charged droplet on the negative friction layer. Upon sliding of the droplet, the negatively charged electrode migrates to the next positioned interdigitated electrode, generating an alternating current in the cyclic process. A corresponding schematic representation in Figure 5b of the potential-well distribution illustrates electron transfer from the droplet to the friction layer, which represents the overall mechanism for charge transfer between tribopairs.

**Figure 5:**
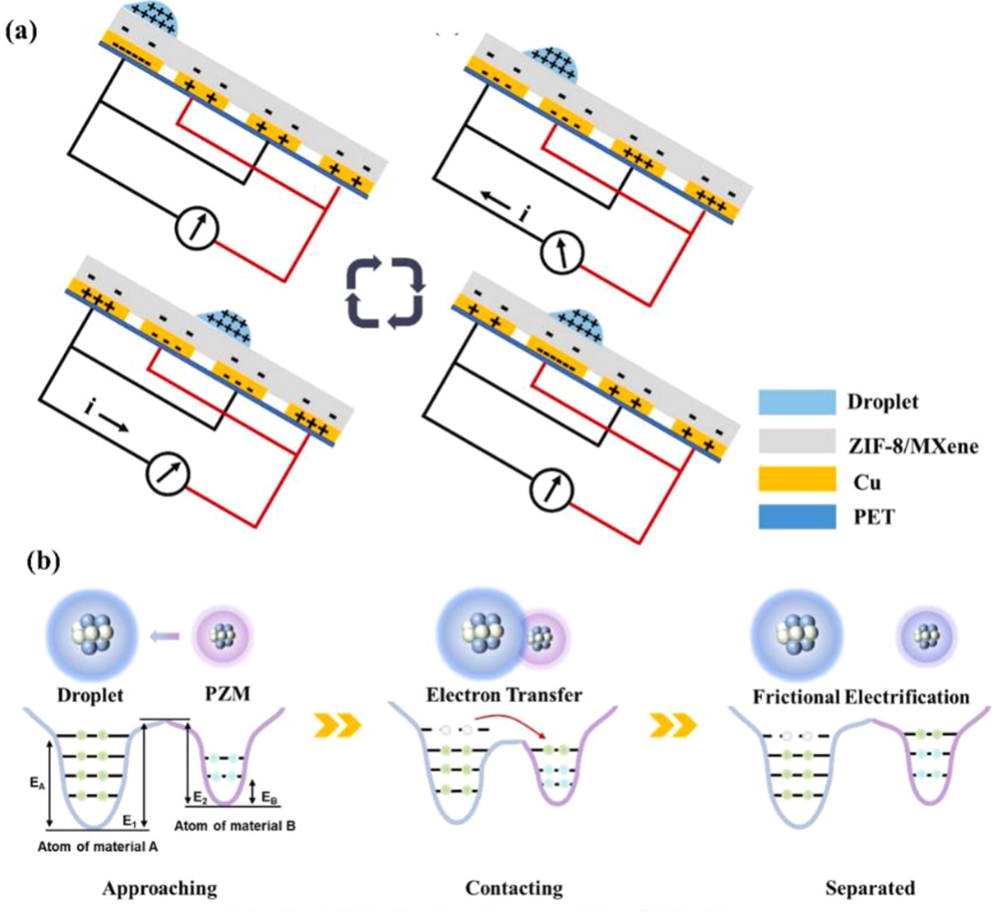
(a) General scheme for AC generation using a modified PZM tribolayer in contact with the droplet and (b) the electron cloud potential for the resulting TENG with the corresponding charge transference between tribopairs. Reprinted from Chem. Eng. J., 506, M. Wang, X. Wang, Y. Nan, H. Zhou, H. Xu, Enhanced of ZIF-8 and MXene decorated triboelectric nanogenerator for droplet energy harvesting, 160137, Copyright (2025), with permission from Elsevier.

On the other hand, the carbon single layers of graphene-based structures are rich in carboxyl and hydroxyl groups, which are relevant binding sites for fixing MOFs^85^ characterizing an important growth substrate for the production of GO-supported core–shell MOFs,^15^ making a wide range of interactions with MOFs,^86^ which are favored by graphene properties such as good conductivity, high hydrophobicity, high stability, and high specific surface area^87^ with implications on the efficient charge separation and high density of charge trapping sites.^15^ In addition, the hydrophobicity of GO inhibits water absorption in the resulting composite, improving stability in high-humidity environments.^85^ As an alternative, Patil et al.^88^ reported that the production of composites based on graphene quantum dots provides a superior performance for the TENG device due to the enhancement in the electron mobility that improves the charge transfer rate at friction conditions.

### 2.4 MOF-Based Modified Flexible Electrodes for TENGs

Despite being considered a standard high-voltage energy source with a high input impedance, the performance of the standard TENG depends on its charge transfer capability and the internal charge-recombination rate. Efforts in the literature have focused on surface and bulk modifications of active tribolayers in TENGs, but many issues remain to be addressed, including the harsh environmental conditions that severely affect the TENG performance. These efforts also include modifying the electrodes since contact deficiencies in charge transfer are a critical issue in conventional electrodes.^50^ In particular, this process is critical for use in wearable TENGs that require truly flexible and integrated electrodes. With this aim, several strategies have been elaborated: Rahman et al. proposed the assembly of a stretchable electrode based on LiCl electrolyte in which ZIF-8 is applied as a reinforcement nanofiller with an additional role as a mechanical reinforcement component, with an increase of 2.7 times greater stretchability than pure hydrogel (the assembled electrode is coated by a protective layer of silicone elastomers^89^).

It is worth mentioning that the modification of electrodes for flexible TENGs is not restricted to MOF-based compounds but considers a diversity of materials, such as conducting polymers (polypyrrole^90,91^ and polyaniline^92^), which offer advantages oversimple chemical polymerization for impregnation of the textile, resulting in high conductivity (good transport properties) and preserved intrinsic mechanical properties of the textile.^93–95^

Alternatively, Wang et al.^50^ proposed the development of flexible electrodes composed of a coating layer of Ecoflex impregnated with carbon powder and MXene, while Zhou and co-workers^96^ prepared an electrode layer by mixing a suspension of MXene and cellulose nanofibers. Other classes of materials used to modify electrodes for TENGs include alternative conductive fillers and supporting matrices, such as carbon black and bacterial cellulose.^97^

The diversity of strategies for modifying MOF-based materials for tribopairs and electrodes results in devices with different performances, as summarized in Table 1, which presents the short-circuit current. open circuit voltage, and power output of the resulting TENGs.

**Table 1:** Electrical Output Performance of MOF-Based TENGs

structure	MOF role	*I*_SC_	*V*_OC_	power density	ref
Nanocrystalline MOF	triboelectric positive layer	55.32 μA	451.80 V	2451.04 mW/m^2^	^13^
MOF nanoflakes embedded in silk fibroin	triboelectric positive layer	8 μA	120 V	263 μW/cm^2^	^14^
Core–shell ZIF-8@ZIF-67 MOF anchored on graphene oxide filler	nanofiller for Ecoflex-based device	47.8 μA	327.7 V	5.75 W/m^2^	^15^
Isoreticular MOF in cellulose-nanofibril	functional filler	0.51 μA	87.6 V		^16^
Impregnated MOF into the fabric	tribopositive fabric	18 μA	110 V	0.625W/m^2^	^71^
Dendrite Co 2-methylimidazole MOF	triboelectric positive layer	4.5 μA	144 V		^98^
Zeolitic imidazole framework −8	triboelectric positive layer	7 μA	164 V		^68^
ZIF-8 nanofiller in a hydrogel	hydrogel encapsulated in the silicone layer of TENG	56.3 mA m^–2^	232 V	3.47 W/m^2^	^89^
UiO-66–4F with Zr_6_O_4_(OH)_4_ and TFA ligants	modified PDMS layer	30.6 μA	937.4 V	38.7 W/m^2^	^53^
Alpha-cyclodextrin- MOF/ Teflon	tribopositive layer	1.2 μA	152 V		^99^
ZIF family/ kapton	tribopositive layer	1.1 μA	60 V		^73^
MOF5	tribopositive layer	2.9 μA	27 V	5.76 μW	^5^
MOF-FCF/PTFE	coating MOF on flexible conductive filler (FCF)	7 μA	47 V	1.1 mW/m^2^	^72^
Co-NPC incorporated PVDF	tribonegative layer	248.66 mA/m^2^	948 V	19.24 W/m^2^ at 95% RH	^65^
PDMS-UiO-66-NO_2_	tribonegative layer	17.3 μA	191 V		^58^
MOF808@MXene TENG	tribopositive layer	30.7 μA	296.22 V	67.4 mW/m^2^	^47^
MIL-88A	tribopositive layer	2.2 μA	80 V		^60^
HKUST-1 on a copper electrode	tribopositive layer		98.8 V	771.8 mW/m^2^	^70^
Graphene quantum dots@MOF-5	tribopositive layer	84 μA	885 V	2971.8 μW/cm^2^	^88^
–OH modified MOF on polyurethane matrix	tribopositive layer	0.47 μA/cm^2^	197.6 V		^59^
MIL-53 impregnated sponge	tribopositive layer	0.9 μA	44.2 V		^100^
MIL-88 A and BaTiO_3_ in PDMS	tribonegative layer	160 μA	486 V	5.82 W/m^2^	^101^
MOF-303	tribopositive layer	85 μA	435 V	7.4 W/m^2^	^102^
ZIF-8@ZnO	tribopositive layer	41.5 μA	200.5 V	0.8 W/m^2^	^103^
ZIF-67/PVA composite	tribopositive layer	185 μA	416 V	7.18 W/m^2^	^104^
MIL-125(Ti)-NH_2_	tribopositive layer	130 μA	512 V	6.75 W/m^2^	^105^

As can be seen, the modification provided by MOFs in TENGs results in a wide range of variation in the voltage output (from 47 to 948 V)—including high power output performance at severe conditions of humidity (95%RH) with a more restricted variation in the current (in the order of μA), characterizing a typical limitation for the fast and continuous charge transference from TENGs to energy charge devices. This process is combined with a high output impedance of TENG (in the range of 10–100 MΩ), limiting the direct driving of conventional electronic devices due to the low conversion efficiency.^106^ To circumvent these limitations, several strategies have been considered, ranging from the integrated assembly of capacitors^107^ until the use of power management circuits (PMCs) to optimize the output impedance of the TENG. These results reinforce the importance of self-charging strategies for practical applications, such as IoT and portable electronics.

## 3 Development of Self-Charging Power Systems Integrated with TENGs

Developing flexible self-charging power systems (SCPSs) by integrating flexible TENGs as active power sources operating in the low-frequency region requires a typical electrical treatment, since the irregular, AC-based output is not compatible with standard storage electronic devices and thus necessitates rectification via a full-bridge rectifier.^108,109^ The production of self-charging energy storage assemblies appears to be a promising trend that can reduce the reliance on external components for signal treatment, thereby enabling faster charging rates.^110^

As a standard energy storage device, a supercapacitor combines electrical double-layer capacitance with pseudocapacitance^111–113^ when configured as symmetric and asymmetric assemblies, with promising applications in bioelectronic devices.^114^ Also, intrinsic and valuable properties of MOFs have been incorporated into SCs when combined with conducting polymers and carbon materials,^115^ enabling their use in all-MOF devices by integrating MOF-based TENGs and MOF-based SCs with minimal electronic devices to process the harvested signal. With this aim, Shrestha et al.^116^ proposed an integrated device, summarized in Figure 6a­(i,ii) and b­(i,ii), in which the charge carriers are generated at a metal-semiconductor heterojunction formed by an Al metal layer and a ZIF-67/PEDOT: PSS polymeric layer, resulting in an increase in output attributed to the ZIF-67 activity. The direct sliding of Al over the semiconducting structure deposited on SEBS imprinted laser-induced graphene (LIG) layer favors the direct transfer of charge via a triboelectric mechanism, as follows: under contact of the Al layer and ZIF-67/PEDOT: PSS, electrons from the Fermi level of the Al migrate into to occupy the lower-energy unoccupied states. Then, the Al becomes positively charged, while ZIF-67/PEDOT: PSS becomes negatively charged, reaching alignment at the Fermi level. At equilibrium, no current is observed (as expected), but when the Al layer slides, frictional energy generates nonequilibrium charges that create electron–hole pairs that move in the direction of the field, thereby generating a DC current characteristic of the tribovoltaic effect.

**Figure 6:**
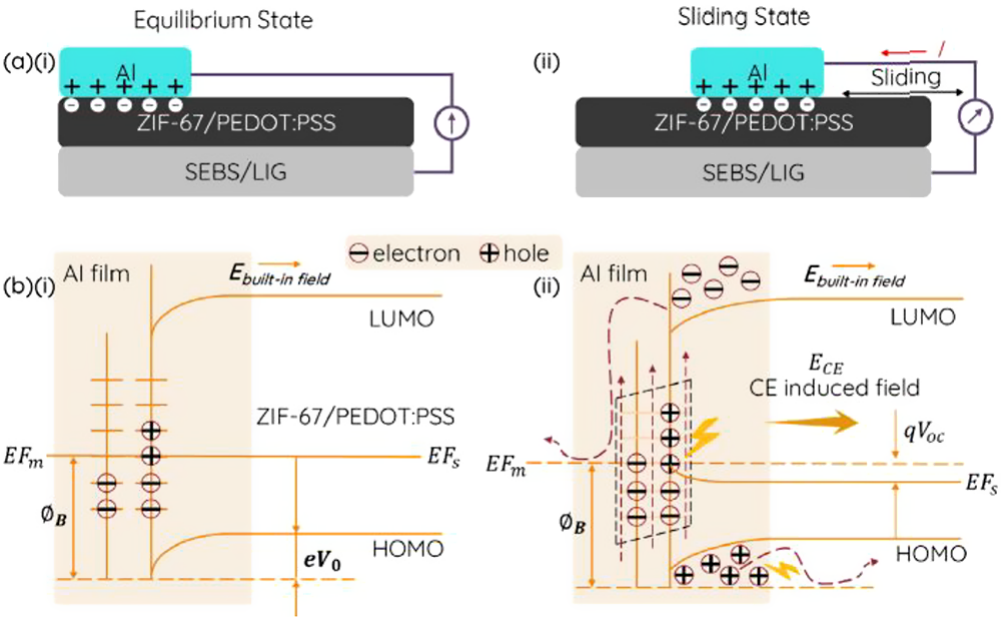
Schematic representation of (a) accumulated charges at the interface between Al layer and ZIF-67/PEDOT: PSS (i) and the generated current under sliding (ii); (b) Corresponding energy band diagram of the Al-ZIF-67/PEDOT: PSS, at the equilibrium state (i) and the sliding state (ii). Reprinted with permission from K. Shrestha, S. Sharma, G.B. Pradhan, T. Bhatta, M.S. Jo, J.Y. Park. MOF-Decorated DC-Tribovoltaic Nanogenerator Integrated with Self-Charging Supercapacitor for Smart PC Screen Time Management. *Adv. Energy Mater.* Copyright [2026] John Wiley and Sons.

Alternatively, Zhang et al. developed the synaptic structures of microsupercapacitors produced by electrospinning and laser engraving, which were embedded in a gel with ZIF-8@MXene/carbon nanofibers as electrodes.^117^ The efficiency in the process of energy harvesting/ storage in microsupercapacitors is controlled by strategies involving power management units that reduce the output voltage and increase the current output—power is the same in a transformer—enhancing the charging rate in the supercapacitor due to the controlled output impedance of the TENG. The authors reported an average charge rate that was 14.5 times higher than that obtained for a pair (TENG/SC) connected via a standard rectifier. Another option is the development of a triboelectric-driven rectification-free device based on the possibility of truly integrating a self-charging supercapacitor power cell (SPC), exploring the so-called tribo-electrochemical mechanism.^118^ The SPC is composed of a P­(VDF-HFP) polymer separator impregnated with an ionic liquid electrolyte, in which a MOF-derived cobalt/LIG/Cu electrode is immersed. The electrode production (Figure 7a) and overall view of the assembled device with an upper and a lower TENGs with a supercapacitor sandwiched between them (Figure 7b) operate under compressive forces, in which the ions migrate in the direction to the electrode layer in SC to be connected to small electronic devices, see the scheme in Figure 7c.

**Figure 7:**
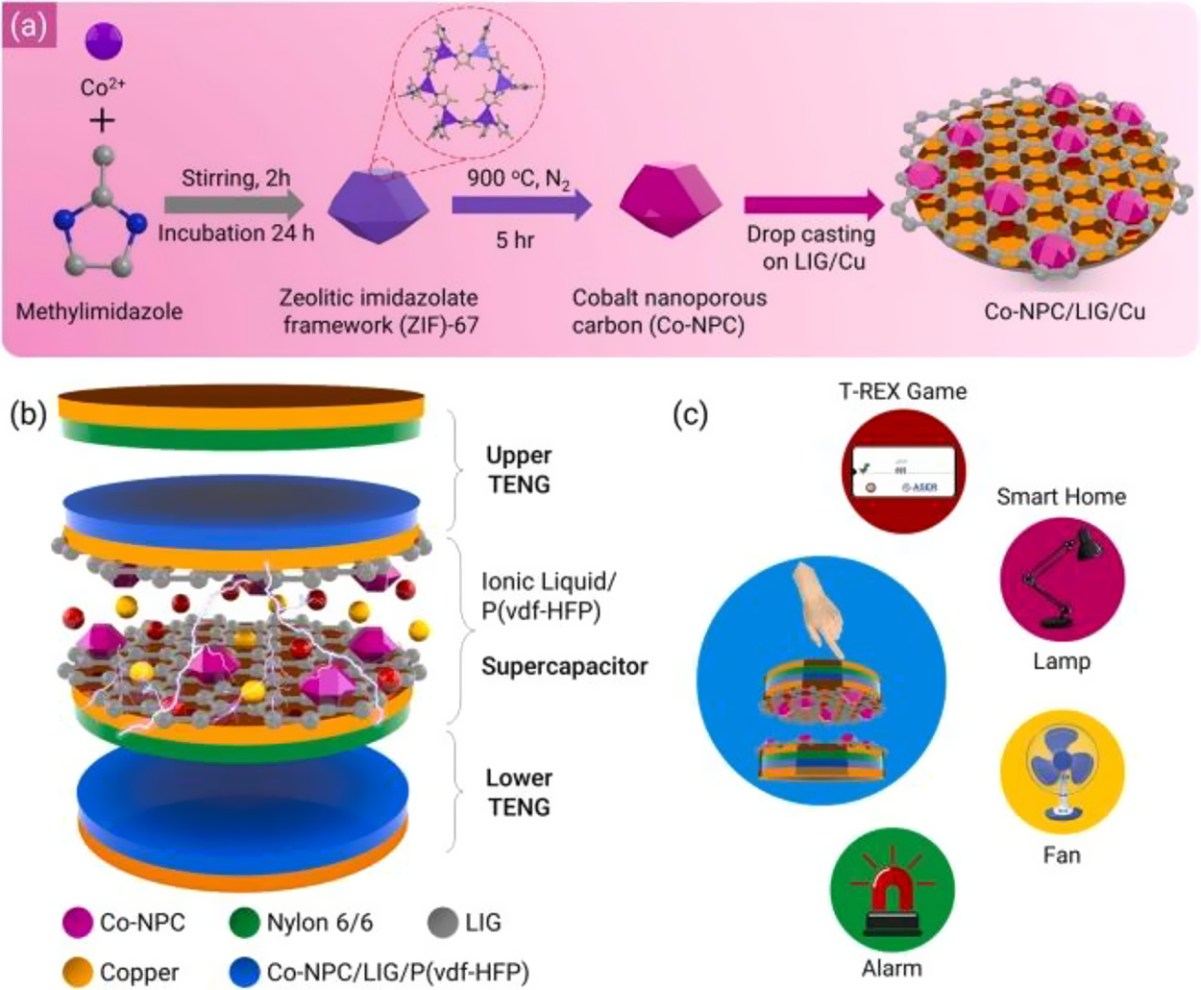
General scheme for the production of the MOF-based electrode (a), representation of the self-charging process of supercapacitors (b) and corresponding applications (c).^118^ Reprinted from Nano Energy, 102, K. Shrestha, S. Sharma, G.B. Pradhan, T. Bhatta, S.S. Rana, S. Lee, S. Seonu, Y. Shin, J.Y. Park, A triboelectric driven rectification free self-charging supercapacitor for smart IoT applications, 107713, Copyright (2022), with permission from Elsevier.

These results illustrate different strategies implemented to optimize the direct conversion of mechanical energy into DC output for electronic devices, presented in an integrated view using PMCs to control the output current/voltage or eliminate rectifiers in the direct generation of a DC signal. In all cases, the direct conversion of movement to a DC value is a valuable step for applications that depend on self-charging units.

As observed, the traditional method of connecting the harvested signal from TENGs via a full-bridge rectifier fails due to the high power consumption of the bridge,^105^ in association with the critical impedance mismatch between TENG and energy storage device, dramatically affects the energy conversion efficiency (while the maximum output power is reached for TENGs using loading resistance in the range of 10^5^–10^7^ Ω, the impedance of batteries and supercapacitors is in the range of 10^–2^–10^2^ Ω).^109^ As a result, the charging rate for standard energy storage devices,^117^ considering different SCPS, varies from 0.17 to 1.6 mV/s. The use of PMCs returns the charging rate to values on the order of 26 mV/s.^117^ In terms of the efficiency of the stored energy, an adequate choice of PMC results in outstanding values in the order of 70–85%.^119,120^

## 4 Conclusion

The porous structure and intrinsic properties of MOFs offer adequate conditions to improve the performance of integrated devices (TENGs and supercapacitors) by enhancing the charge-transfer rates, charge trapping, hydrophobicity, and operation under harsh conditions. The integration of energy harvesting and energy storage in all-MOF devices depends on additional strategies for pretreating the electrical output at interfaces, such as current amplification, impedance tuning, or standard signal rectification. Alternatively, the use of electrodes based on encapsulated hydrogels minimizes the required circuit for treatment in rectifier-free self-powered systems. In addition to these advanced strategies for energy harvesting and use, MOFs serve as powerful nanofillers that offer advantages in dielectric response, surface porosity, and operation under extreme humidity conditions through effective interactions with nanostructures (graphene oxide, carbon nanotubes, conducting polymers, and MXenes).
